# A Single Polyaniline Nanofiber Field Effect Transistor and Its Gas Sensing Mechanisms

**DOI:** 10.3390/s110706509

**Published:** 2011-06-24

**Authors:** Dajing Chen, Sheng Lei, Yuquan Chen

**Affiliations:** College of Biomedical Engineering, Zhejiang University, Hangzhou, 310027, China; E-Mails: djchen@zju.edu.cn (D.C.); thunder@zju.edu.cn (S.L.)

**Keywords:** polyaniline, single nanofiber, field effect, NH_3_ sensor

## Abstract

A single polyaniline nanofiber field effect transistor (FET) gas sensor fabricated by means of electrospinning was investigated to understand its sensing mechanisms and optimize its performance. We studied the morphology, field effect characteristics and gas sensitivity of conductive nanofibers. The fibers showed Schottky and Ohmic contacts based on different electrode materials. Higher applied gate voltage contributes to an increase in gas sensitivity. The nanofiber transistor showed a 7% reversible resistance change to 1 ppm NH_3_ with 10 V gate voltage. The FET characteristics of the sensor when exposed to different gas concentrations indicate that adsorption of NH_3_ molecules reduces the carrier mobility in the polyaniline nanofiber. As such, nanofiber-based sensors could be promising for environmental and industrial applications.

## 1. Introduction

Conducting polymers have attractive features such as mechanical flexibility, ease of processing, and modifiable electrical conductivity [1,2]. Among these materials, polyaniline (Pani) has been studied for many applications including logic circuit components [3], electromagnetic shielding [4], chemical sensing [5,6] and anticorrosion coating [7] due to its easy synthesis, room temperature operation, and relative environment stability [8,9]. Conductive polymer-based devices are fabricated and studied as field effect transistors (FETs), since they form the basic unit in digital circuits and semiconducting components. The field effect also contributes to the improvement of chemical sensors [10]. In recent years an enormous amount of research has been devoted to the development of organic semiconductors and nanostructures [11,12].

Due to its high toxicity, the detection of NH_3_ in air is desirable in the area of environmental monitoring and industrial process control. Conductive polymer and carbon nanotube (CNT)-based NH_3_ nanoscale sensors have been attracting considerable attention in recent years [13–15]. For many sensor applications in wireless transducer systems and portable devices, ultralow-power consumption will be required. We reported a single polymer fiber sensor which requires much less energy than conventional polymer film and CNT sensors [16].

Although the structure of nanosensors offers the prospect of high sensitivity and rapid response, the incorporation of nanostructures in sensor devices is limited by the difficulty in locating and electrically connecting the nanoscale sensors to the microelectrodes. In this paper, a single polyaniline nanofiber sensor has been utilized based on near field electrospinning [17,18]. We deposited polyaniline nanofibers in a controlled 2-D pattern without using a conventional lithography process. I-V characteristics were measured in order to differentiate the gas sensitivity results from charge transfer between adsorbed gas molecules and the conductive polymer channel or from the gas species induced Schottky barrier at the polymer-electrode contact.

As a single nanofiber sensor, the signal-to-noise ratio is influenced by the ultra low working current [19]. The field effect can be an advantage for nanofiber sensors if the sensitivity of a system is limited by the signal-to-noise ratio. This sensor reaches a NH_3_ sensing concentration under 1 ppm with a response time of less than 10 s. Results indicated the NH_3_ sensor has the advantages of sensitivity, reversibility and dynamic character.

## 2. Experimental Section

Polyaniline nanofibers were synthesized by near field electrospinning. We investigated the effect of the composite content on the fiber morphology and properties. A 0.6:1 mole ratio of camphorsulfonic acid (HCSA) and polyaniline was dissolved in chloroform to 1.5 wt% concentration. The solution was magnetically stirred at room temperature. The resulting solution was filtered through a 0.45 μm syringe filter and 0.5 wt% polyethylene oxide (PEO) were added to the solution as plasticizer and stirred for an additional 2 h.

**Figure 1.**
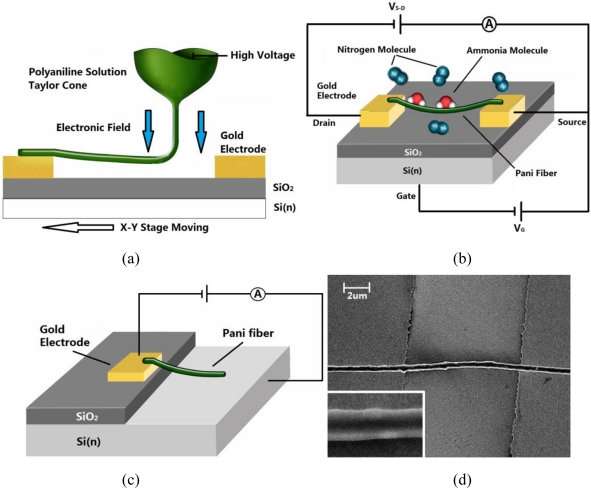
(**a**) Near field electrospinning and device schematic. (**b**) Structure of device A. (**c**) Structure of device B. (**d**) SEM of single Pani fiber across two electrodes. (Inset) Higher magnification view of the single Pani fiber.

A schematic depicting the working principle of the electrospinning process is shown in Figure 1(a). About 0.5 mL of solution was placed in a hypodermic syringe, and the needle was connected to 0.8 kV with respect to the grounded counterelectrode situated about 2 mm from the tip of the needle. As electric forces on the polymer droplet at the end of the needle overcome the surface tension, a jet is issued forth. The substrate was moved in a computer controlled trajectory at a rate of 30 mm/s. By applying different trajectories and speed, the quantity and position of the fibers can be controlled.

As illustrated in Figure 1, two devices with different structures were employed in our experiments: (1) Pani fiber contacts with two gold electrodes (device A). A schematic of device A in an ambient N_2_ environment with NH_3_ vapor molecules adsorbed on the fiber surface is shown in Figure 1(b). (2) Pani fiber, marked as device B, was also deposited on a gold electrode and n-doped silicon as shown schematically in Figure 1(c). Figure 1(d) presents a scanning electronic micrograph of a single polyaniline fiber sensor lying across two gold electrodes. The average diameter of the fiber is 400 nm. The fiber length between two electrodes is 10 μm. In the gas sensitivity measurements, the current through the sensor was monitored at a fixed applied voltage. The sensor was positioned in a sealed chamber with gas flow inlet and outlet ports. Dry nitrogen gas was used as background gas during all the tests.

## 3. Results and Discussion

**Figure 2.**
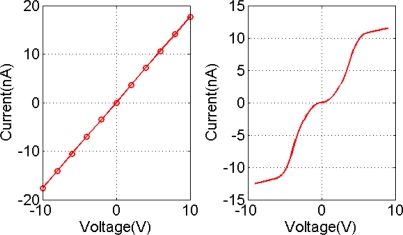
I-V characteristics of device A (**left**) and device B (**right**).

Figure 2 shows the corresponding I-V curve for two devices. A linear response of device A confirms that the polymer contact with the gold electrodes is Ohmic. When the positive terminal of voltage supply was connected to the doped silicon terminal for device B, the forward biased diode response confirms the formation of Schottky barriers at the polymer and n-doped silicon contact. Device A is used to study the gas absorbance influence on the field effect of conductive polymer channel.

**Figure 3.**
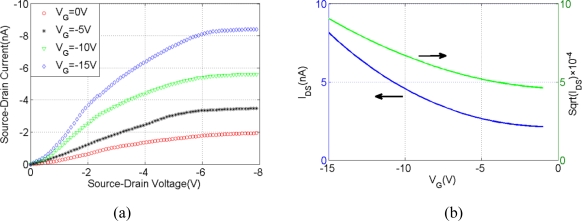
(**a**) Output characteristics of single Pani fiber. I_DS_ *versus* V_DS_ for four different gate voltage (V_G_). (**b**) Transfer characteristics of single Pani fiber. I_DS_ and I_DS_^1/2^ *versus* V_G_ for V_DS_ kept at 10 V.

Figure 3(a) shows the output characteristics of the device under different gate voltages (V_G_). The source current (I_S-D_) curve demonstrates saturation at drain voltage (V_S-D_) < −6 V and the magnitude of the saturation current was controlled via the application of V_G_. I_S-D_ increases with increasing V_G_ demonstrates that the device operates as a FET, and also that the majority carriers are holes, as expected for doped polyaniline [20].

The field effect hole mobility μ is calculated by equation:

(1) $${\text{I}}_{\text{DS}}=\frac{\text{w}}{2\text{L}}{\text{C}}_{\text{i}}\mu {\left({\text{V}}_{\text{G}}-{\text{V}}_{\text{TH}}\right)}^{2}$$

where V_TH_ represents the threshold voltage, W and L correspond to the fiber diameter and length respectively. C_i_ is the gate dielectric capacitance per unit length.

The capacitance C_i_ can be calculated as:

(2) $${\text{C}}_{\text{i}}=\frac{2\pi ɛ{ɛ}_{0}\text{L}}{\text{ln}(2\text{h}/\text{r})}$$

where r represents the radius of the fiber, ε_0_ is the permittivity of free space, h and ε represent the thickness (200 nm) and average dielectric constant of the device, respectively [21]. Using these figures, we get the hole mobility μ to be 2 × 10^−3^ cm^2^/Vs. In Figure 3(b), the square root of the I_S-D_ is on a straight line for high drain voltage. The square root of I_S-D_ *versus* V_G_ is extrapolated to the V_G_ axis to obtain the threshold V_TH_ (−3 V).

**Figure 4.**
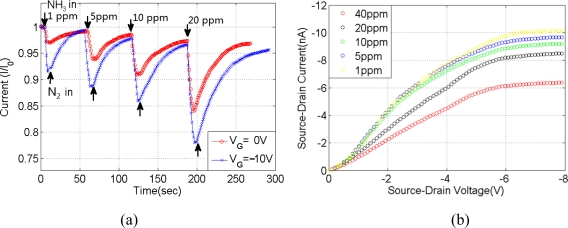
(**a**) Real-time response to different concentrations of NH_3_ at V_G_ = 0 V and V_G_ = −10 V. (**b**) FET output characteristics in different NH_3_ concentrations at V_G_ = −5 V.

A constant 5 V potential was applied on the source and drain electrodes for the real-time monitoring of the Pani fiber’s response to NH_3_. The time-dependent current responses of the Pani fiber upon exposure to different concentrations of NH_3_ gas in a nitrogen environment are shown in Figure 4(a). Under 0 V gate voltage, the sensor’s working current decreased 2.7% when exposed to 1 ppm NH_3_ gas flow. In contrast, a much greater current decrease of 7% for 1ppm NH_3_ is obtained under V_G_ = −10 V. After pump nitrogen into chamber, the sensor recovers to more than 90% of original resistance in minutes. Device With −10 V gate voltage also showed greater current decrease when it was exposed to 5, 10 and 20 ppm NH_3_. The current noise level during test is below 0.1 nA. Considering the noise to signal ratio, the limit of detection is below 0.5 ppm NH_3_.

Figure 4(b) shows the FET output characteristics of a Pani fiber sensor upon exposure to various NH_3_ concentrations. The electrical characteristics of Pani single fiber were studied by monitoring I_DS_ while sweeping V_DS_ at a fixed value of V_G_ = −5 V. The effect of NH_3_ molecule on Pani fiber is taking up protons from Pani, thus forming NH_4_+ and reducing carrier concentration. The sensor still follows the field effect characteristics while hole mobility μ decreased to 1.5 × 10^−3^ cm^2^/Vs when exposed to 10 ppm NH_3_.

**Figure 5.**
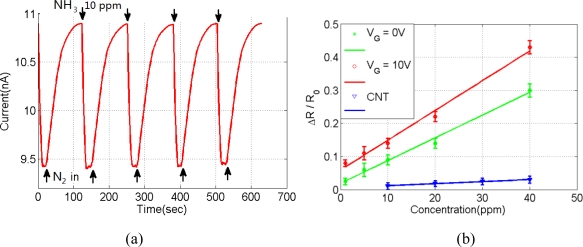
(**a**) Repeatability test with 10 ppm NH_3_ (V_G_ = −10 V). (**b**) Sensitivity compare of Pani fiber sensor with or without gate voltage and CNT sensor.

Devices have been cycled up to five times and good resistance reversibility can be observed from Figure 5(a). Each cycle was carried out in 10 ppm NH_3_ and nitrogen after saturation occurs in the presence of either gas. The extracted sensitivities, S = ΔR/R_0_, of the Pani nanofiber under two gate voltages and CNT sensor are shown in Figure 5(b). Each data point in the plot is collected by five repeated measurements for the error estimation. Sensitivity improved when the gate potential was increased from 0 V to −10 V, as can be seen from Figure 5. This improvement indicates that the single Pani fiber sensor offers tunable sensitivity with field effect. This test result is compared to our previous work on CNT dielectrophoresis deposition. Sensitivity is improved while current consumption decreased from the milliampere level to microamperes [16]. Decreased working current made the sensor suitable for applications in portable devices and the FET structure will enhance the signal-to-noise ratio when the working current is too low.

The single fiber FET structure places the gate and dielectric below the active layer. The top-contact and bottom-gated structure contributes to the simplification of the deposition procedures. Also, the semiconductor character of Pani fiber enables field effect enhancement of the sensor response. The gate dependence of NH_3_ detection sensitivity can be attributed to the gate modulation of the carrier concentration in a nanofiber channel. The field effect transistor has the ability to amplify the signal and to gate-modulate channel conductance, so the sensitivity can be improved by adding a suitable gate voltage. The use of an insulating substrate and individually patterned gate allow the device parameters to be independently controlled and tested. Conductive nanofibers could be used as sensor arrays capable of detecting multiple chemicals due to the nano scale and ultra-low working current [22].

## 4. Conclusions

We have demonstrated a simple method for creating single nanofiber gas sensors integrating microfabricated electrodes and FET structures. Sensing mechanisms for a polyaniline based single fiber NH_3_ sensor have been systematically studied on a FET platform. I-V characteristic curves of the sensor show evidence of current saturation for V_S-D_ voltage biases. The dominant gas sensing mechanism of the Pani fiber is deprotonation of Pani by NH_3_, resulting in a resistance increase. The sensor exhibits high sensitivity (7% *versus* 1 ppm of NH_3_) with good tunability under appropriate gate voltages.
